# Conservative management of fifth metacarpal head fracture in a professional cricketer: A case study and literature review

**DOI:** 10.1002/ccr3.2960

**Published:** 2020-05-28

**Authors:** Tej Pandya, Susan Dale, Ella Donnison, Stefan Kluzek

**Affiliations:** ^1^ Faculty of Biology, Medicine and Health University of Manchester Manchester UK; ^2^ Medical Department England and Wales Cricket Board London UK; ^3^ Nuffield Department of Orthopedic, Rheumatology, and Musculoskeletal Sciences University of Oxford Oxford UK; ^4^ Centre for Sports Medicine School of Medicine University of Nottingham Nottingham UK

**Keywords:** emergency medicine, orthopedics, sport medicine

## Abstract

This is a unique presentation of common injury in a young professional athlete. The sensitivity of typical acute clinical signs in athletes is not known. Adequate splinting and early graded mobilization are key for successful rehabilitation, which was shorter than reported in the literature.

## Graphical abstract

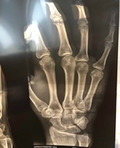

## 1 INTRODUCTION

Diagnosis of fifth metacarpal head fracture with collateral ligament injuries can be difficult. We present a case of a professional cricketer with a fifth metacarpal head fracture, who played a match before pain made the injury obvious. Following immobilization, she returned to baseline within six weeks using nonsurgical management.

The traumatic fifth metacarpal (5th MC) fractures and collateral ligament injuries are often caused by punching injury or a direct blow from a fall or crush injury. They are common upper extremity injuries in adults, and fractures of the metacarpal are the most common type of hand fractures accounting for up to 40% of injuries.^1^ These injuries usually occur in a young, active population and if left inappropriately managed can lead to weakened fifth grip initiation and diminished hand function.^2^ Most typical 5th MC fractures involve shaft fracture (oblique or transverse), and debate about best management is widespread among the literature.^3^, ^4^

Management of fractures to the metacarpal bones of the hand is decided on both the clinical examination and radiological findings. There is a paucity of clinical research surrounding management of the 5th MC neck fractures and return to sport in professional athletes, despite this being a frequent upper limb injury.^4^ A recent systematic review showed no difference in patient‐reported radiograph or clinical outcomes at 12 months for patients with 5th MCP neck fractures between surgical and conservative strategies, and therefore considering surgical risk, a conservative management strategy may often be optimal.^4^ Here, we present a case in a young athlete, who successfully rehabilitated and returned to sport with conservative management.

## 2 CASE PRESENTATION

### 2.1 Presenting features

**FIGURE 1:**
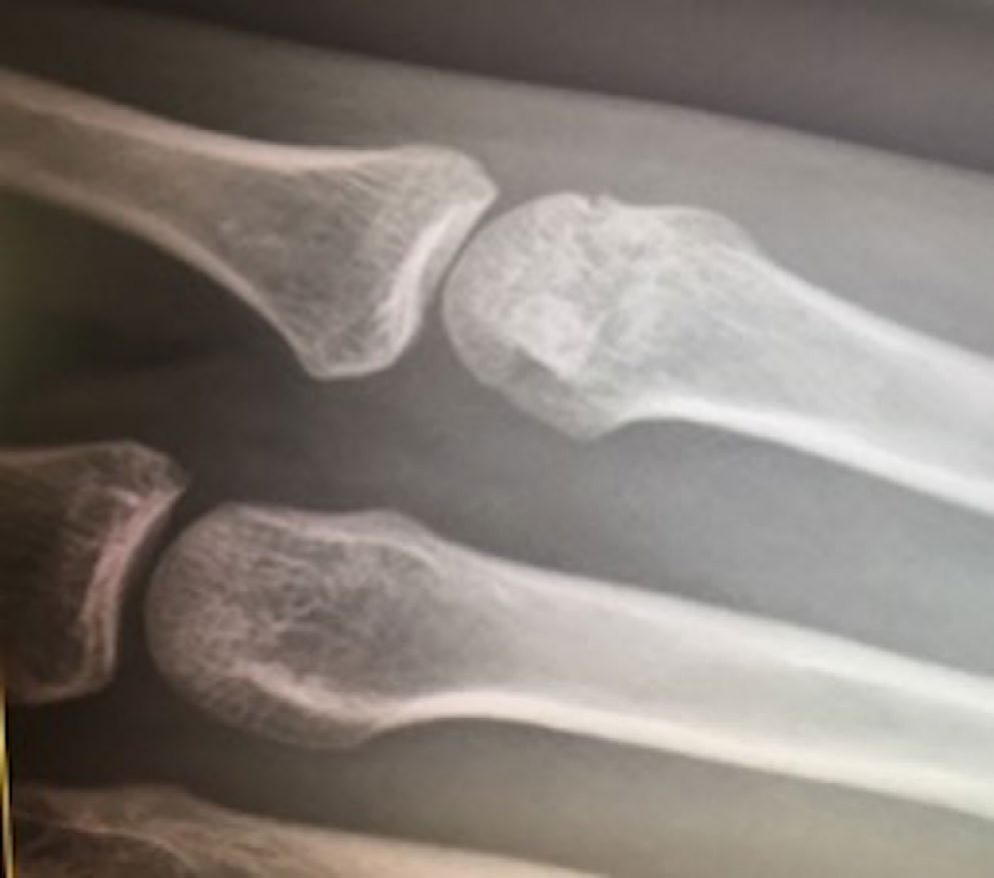
X‐ray of the hand

**FIGURE 2:**
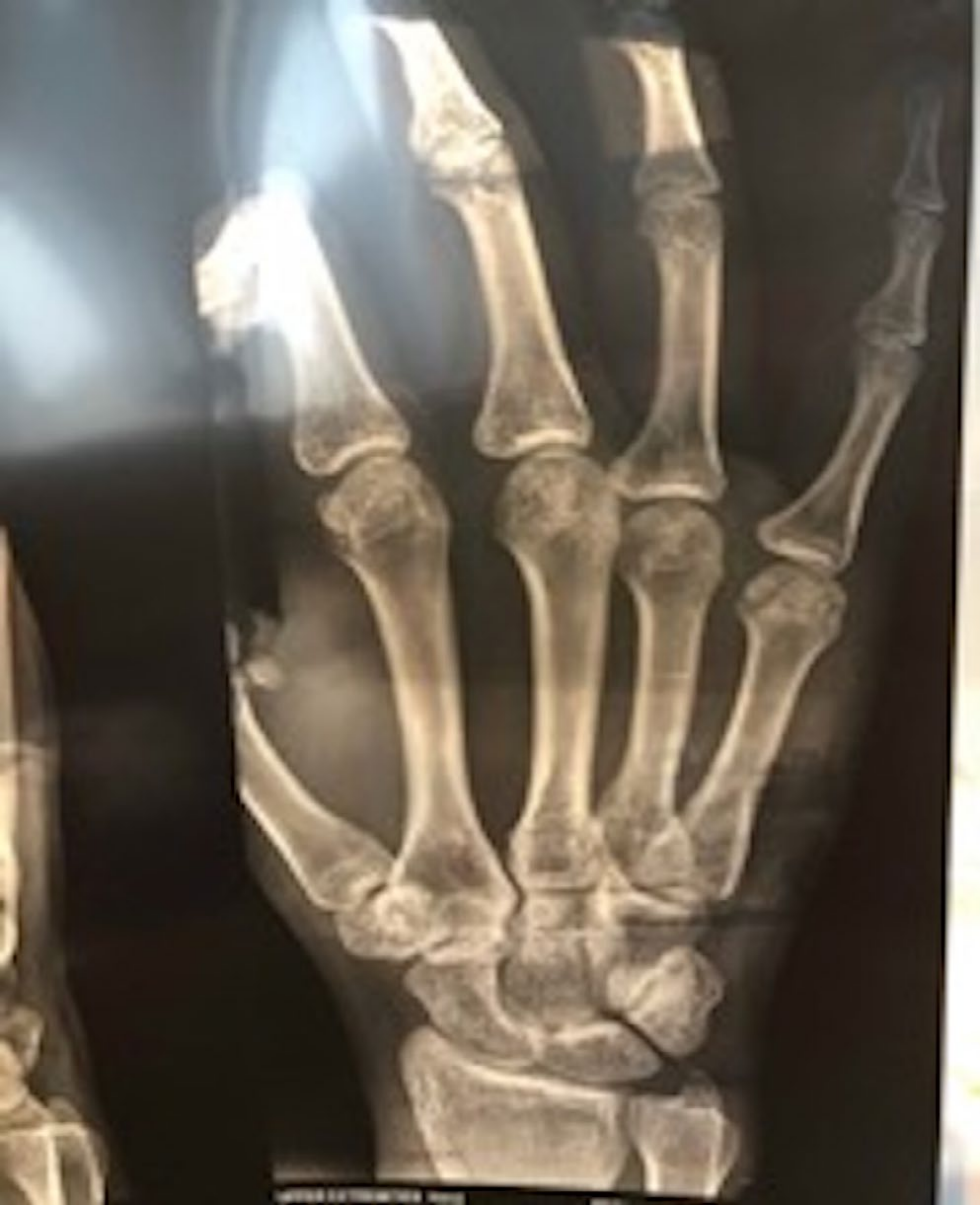
MRI of the hand

We present a case of a fit and healthy 19‐year‐old female professional cricketer who developed nondominant traumatic hand pain while fielding as part of a prematch warm‐up. Her fifth digit stubbed the ground, and she landed on her hand while catching an awkward ball. On initial assessment, the only notable initial symptom was mild metacarpophalangeal joint (MCPJ) swelling, with normal function, no obvious deformity and unaffected neurovascular status. Initial management involved buddy strapping, and the athlete decided to carry on with training and later played in a one‐day international (ODI) game. On subsequent examination, there were negative cascade and flexion cascade signs with a lax collateral ligament and pain on movement. Subsequently, swelling and bruising developed within the first two hours with no significant deterioration of pain but limited flexion. An X‐ray (Figure 1) showed an impacted fracture of the head of the metacarpal, and an MRI (Figure 2) confirmed not only the fracture but also a noncomplete oblique fracture. Following review and discussion with a hand surgeon, a metacarpal thermoplastic splint was given to the athlete (including the wrist and the distal interphalangeal joint). A review of the athlete also took place to optimize their nutrition and to modify any lifestyle risk factors, which could lead to inadequate bone healing.

### 2.2 Medical/social/family history

The athlete had no significant past medical or surgical history.

#### 2.2.1 Treatment

The splint was used for just over 1 week after a hand surgical review. At this point, the immobilization splint was replaced with buddy/ neighbor taping to allow commencement of movement (tendon glides) as MCPJ stiffness is a common problem (Table 1). The process for swelling control included elevation in a resting position, compression with an adhesive bandage (using a spiral application), kinesiology taping, and active range of motion of unaffected joints. We measured power improvement via both a grip and pinch assessment and functional assessment. The grip and pinch assessment involved a hand dynamometer and pinch gauge. It also included utilizing a hand and finger trainer and using a gel resistance exercise ball with increasing number of squeezes. Functional exercises involved trialing cricket‐specific skills such as picking up a ball and throwing a cricket ball to progressively increasing distances.

**TABLE 1:** Physiotherapy treatment and rehabilitation aims

Week	Treatment	Aims
1‐2	* Immobilization in the metacarpal splint * Gentle movement introduced in second week with the use of buddy/neighbor taping	* To make alignment * To prevent stiffness * To control swelling
3‐4	* Range of movement exercises (tendon glides) * Stretching of the MCPJ in order to improve the movement range, for example, squeezing a soft rubber ball and holding for 3 s	* To improve power
5	* Squeeze a soft rubber ball and hold the squeeze for 5 s—2 sets of 15.	* To restore grip strength
6	* Sport‐specific exercise	* To return to play

## 3 OUTCOME AND FOLLOW‐UP

With regular rehabilitation supervised by a physiotherapist, the athlete recovered within four to five weeks. Follow‐up X‐ray showed good congruity of the joint, and clinical assessment showed no increased laxity of the collateral ligaments.

## 4 DISCUSSION

Fractures of the metacarpal head are by definition intra‐articular and are rarer than fractures involving the distal parts of the bone. The second metacarpal is the most common to be injured, and the cause is often as a result of a direct blow or crush injury. Fractures in this region are often comminuted, causing additional difficulty with their management. Surgically managed cases (using open reduction and fixation) have been published before reporting significant improvement in functional outcomes within 12 months.^4^ Indeed, one large case series reported that no formal supervised hand rehabilitation was given to its 54 patients.^3^ Controversy remains regarding the timing and usage of conservative management and ability to come back into sports associated with high‐impact hand injuries such as cricket or boxing. Early work suggested that risks of nonoperative management are deformity.^3^ However, despite a mild cosmetic abnormality being reported, conservative management of these patients has resulted in excellent functional outcome, particularly if there is less than 25% joint involvement.^5^ For collateral ligament injuries associated with 5th MC fractures, surgical repair is required due to persistent laxity.^6^ Literature suggests that in a sporting population, the average return to play is between six and eight weeks.^7^ It recommends the use of continuing splinting for between four and six weeks after injury in patients.^8^ Most literature also recommends exercise initiated within the first week to recover digital motion and to not start strength and conditioning exercises until callus formation is seen on an X‐ray.^7^, ^8^, ^9^ In a sport‐specific population, one review also advocated wearing a splint for the rest of the sporting season to prevent reinjury.^2^ However, It is important to note that majority of the work was performed in nonathletes, and largely looked at metacarpal neck fractures.

Severe acute pain associated with injury is widely recognized as a potential sign of bone injury or fracture. However, it may be less prominent in players used to high‐impact insults to the hand, particularly athletes, who may have increased tolerance to pain by virtue of their profession.^10^ For example, the cascade sign that is widely used to assess the rotational deformity of the fingers is associated with metacarpal and phalangeal malalignment.^11^ Here, fingers converge toward the scaphoid tubercle when flexed at the MCPJ and PIPJ, with a positive test showing altered normal alignment. In this case, however, the cascade sign was negative, and therefore, it is worth noting that the sensitivity of clinical findings may be limited in cases where shaft of the bone is not completed.

In summary, our case demonstrates that conservative management can be effectively utilized to return a competitive athlete to full sporting participation at preinjury standard within six weeks. This is slightly quicker than the timing described in the literature, which is between 6 and 12 weeks. We suggest that conservative treatment may be most appropriate in most instances in the absence of a current consensus and previous systematic review.^4^ Clinicians should be aware of risk factors, injury, pain, and unknown sensitivity of typical clinical signs of this injury. Many questions remain regarding the optimal threshold diagnostic indications and most appropriate treatments for the general population. A high index of suspicion must be maintained particularly when treating young, professional athletes.

## CONFLICT OF INTEREST

The authors wish to declare no conflicts of interest.

## AUTHOR CONTRIBUTION

SK: conceived the idea for the manuscript. SD and ED: were involved in the rehabilitation of the patient. TP and SK: wrote the manuscript, which was then amended by SD and ED. All authors read and approved the final version of the manuscript.
